# Through children’s own lenses: assessing risky play tendencies via a tablet-based interface

**DOI:** 10.3389/fpsyg.2026.1951082

**Published:** 2026-09-22

**Authors:** Neslihan Tuğçe Özyeter, Hatice Gözde Ertürk Kara, Esra Ünlüer, Levent Durdu

**Affiliations:** 1 Faculty of Education, Department of Measurement and Evaluation in Education, Kocaeli University, Kocaeli, Türkiye; 2 Faculty of Education, Department of Preschool Education, Kocaeli University, Kocaeli, Türkiye; 3 Faculty of Education, Department of Educational Sciences, Kocaeli University, Kocaeli, Türkiye

**Keywords:** digital assessment, pictorial scale, play behavior, preschool children, tablet-based assessment

## Abstract

**Introduction:**

Risky play is increasingly recognized as a developmentally significant form of play that supports children’s physical competence, emotional regulation, and risk-management skills, yet capturing children’s own tendencies toward such play remains methodologically challenging. This study addresses this gap by introducing a tablet-based, pictorial assessment tool [the Digitized Willingness to Risk in Play Scale–Pictorial (d-WRiPS-P)] designed to assess preschool children’s risky play tendencies through their own lenses.

**Methods:**

A total of 198 Turkish children aged 5–6 years participated in the study. Both convenience sampling and maximum variation sampling methods were used to recruit participants for the group. In the study, the validity of the d-WRiPS-P was evaluated using CFA with WLSMV estimation, and its reliability was determined using Ordinal Omega;measurement invariance was tested across gender and upper/lower 27% temperament groups, and the scores of the WRiPS-P and d-WRiPS-P were compared using Pearson correlation and paired samples t-test.

**Results:**

This study provides evidence that the d-WRiPS-P is a valid and reliable tool for assessing risky play tendencies in 5–6-year-old children. Results support its three-factor structure, high construct validity, internal consistency, and measurement invarianceacross gender (at the scalar level) and temperamentsubgroups (activity and reactivity at the strict level).

**Discussion:**

The original contribution of this study lies in providing evidence for the validity of a digitized assessment tool that enables 5–6-year-old children to participate independently without adult assistance or literacy requirements, utilizing audio-narrated touchscreen technology. This independent administration offers standardized instructions and automates scoring, which may help promote consistency in data collection while reducing reliance on administrator input. Beyond its application in the Turkish context, the tool provides a standardized and scalable framework with potential for cross-cultural, multilingual studies and practical use by educators and researchers.

## 1 Introduction

Children in the preschool years have a natural tendency to play in environments that challenge them physically and emotionally, to test their limits, and to cope with their fears (Sandseter, 2009a, 2009b). These activities, defined as “risky play,” fall into six universal categories: playing at great heights, high-speed play, using dangerous tools, being near dangerous elements, rough-and-tumble play, and exploratory play in areas where children may wander out of sight(Sandseter, 2007). Although adults’ safety-focused, overprotective approaches may restrict these play areas (Jelleyman et al., 2019; Sicim Sevim and Bapoğlu Dümenci, 2020), recent research shows that risky play environments serve as a critical context for cognitive development where children test their own potential and learn to manage accidents and solve problems (Karabon and Steiner, 2020). According to The Integrative and Socio-Ecological Model, children’s decisions to take risks are not attributable to a single cause, but are shaped by a complex interaction of individual characteristics (cognitive assessment of injury risk, temperament, past experiences, etc.), family factors (parental modelling of risk-taking, encouragement, sibling interactions, etc.), and social/ecological factors (situational contexts, peer pressure, societal cultural beliefs regarding risky play, etc.) (Morrongiello and Lasenby-Lessard, 2007; Karabon and Steiner, 2020). From the moment a child is born, under the influence of all these complex factors, the child develops a unique internal propensity for risk-taking. Accurately assessing this internal propensity for risk-taking in children is important for supporting their holistic development without restricting it. Through accurate assessments, rather than imposing restrictive prohibitions on children identified as having a high risk-taking tendency, hazard awareness training can be provided; playgrounds can be designed according to the “as safe as necessary” philosophy (Cevher-Kalburan, 2014). For children identified as having a low risk-taking tendency, it is possible to prevent them from being deprived of the developmental opportunities offered by risky play, such as problem-solving and emotional regulation. Children can be introduced to gradual risks that they can control (Brussoni et al., 2012). The results obtained through such objective measurements can help adults eliminate overprotective or unnecessary interventions in children’s play by highlighting the difference between the “risk the child seeks” and the “risk the adult permits” (Di Norcia et al., 2018).

Developed to measure children’s innate tendencies from their own perspectives rather than through indirect adult reports, the Willingness to Risk in Play Scale: A Pictorial Scale for Preschool Children (WRiPS-P) is a valid and reliable direct assessment tool that addresses this need in the literature. The scale assesses children’s tendencies in three key categories of risky play “disappearing/distancing,” “physical activity,” and “high speed/dangerous tools” through 15 items. Instead of human figures, the illustrations feature a cartoon cat character; this ensures that children can make decisions safely, without being influenced by biases related to gender, race, or ethnic origin. Through this cat character, children choose which cat they would like to be from among four illustrated options ranging from not wanting to take any risks (Level 0) to wanting to take a high level of risk (Level 3). For example, an item that measures children’s tendency to wander off or drift away (becoming independent from adults) uses a scenario involving a cat exploring the market. In this item, the child selects one of the following four pictures based on their own inclination: exploring the market while holding an adult cat’s paw (Level 0—no risk), exploring while the adult cat is nearby (Level 1: low risk), exploring while the adult cat is far away but still within sight (Level 2: moderate risk), and finally, exploring entirely on its own in a place where the adult cat cannot see it (Level 3: high risk). WRiPS-P is a paper-and-pencil, illustrated assessment that allows children to express their levels of risk-taking through a familiar cartoon character, without being hindered by language barriers or cognitive strain. Although the scale allows children to reflect their risk-taking tendencies from their own perspective, the nature of the instrument requires an adult (researcher or teacher) to read the instructions and guide the process (Özyeter et al. in review).

### 1.1 Methodological exploration: direct measurement of variables and digital autonomy

The ability to accurately capture risk-taking propensity (central to this research) depends heavily on the objectivity of the measurement method. Roepstorff et al. (2025) point to a significant misalignment between indirect assessment tools based on parent and teacher reports and children’s direct performance outcomes, tracing this mismatch to adults interpreting children’s behavior from general, superficial reactions and falling prey to overestimation bias. This implicit bias (Yurt and Keleş, 2019) leads children, left to their own devices, to choose the “allowed risk” they see permitted rather than the “desired risk” they actually want (Di Norcia et al., 2018). The same social-desirability pressure can affect paper-and-pencil instruments administered under adult supervision, such as WRiPS-P (Özyeter et al., in review), making it essential to develop direct measurement methods that let children express their decisions free from adults’ biased observations and from children’s own tendency to seek approval (Di Norcia et al., 2018; Plowman, 2016).

The tablet-based tool developed in this study retains the logic of the WRiPS-P illustrated scenarios while changing the format of implementation: the child listens to the instructions via voice-over, visually examines the four risk levels associated with each scenario on the screen, and indicates their choice by touching the screen directly. After handing the device to the child, the adult does not intervene in the process (either verbally or through behavior) does not look at what the child is selecting, and provides assistance only in the event of a technical glitch. As it stands, the tool transfers the roles of reading instructions and guiding the process (which an adult typically assumes in the pen-and-paper version) to the interface, allowing the child to make choices independently of the adult’s immediate reactions.

This tablet-based tool has been designed with the motor and attention limitations of early childhood in mind to facilitate the child’s interaction with the screen and reduce measurement errors. To prevent “target-miss” errors, which frequently occur due to weak fine motor skills in the preschool years, the tool excludes “drag” tasks and uses only the “tap” format (Mansor et al., 2008). The pace of the test is entirely up to the child (Cameron et al., 2024). After the child makes a selection, the “Next” button allows them to confirm their answer and navigate back to previous items as desired, with the goal of methodologically eliminating the impulsive errors and unintentional touches that are frequently observed in children (Mansor et al., 2008; Roepstorff et al., 2025). In addition, the tool includes a pre-test demo phase. Although there are criticisms in the literature regarding whether tablet use is influenced by prior device experience (digital familiarity), research has shown that, thanks to the pre-test demo (practice) phase, even preschool children who have never used a smart device before can successfully interact with touchscreens, and that a lack of digital experience does not result in a significant difference in performance (Mukherjee et al., 2020). Taken as a whole, these interface design decisions support the tool’s “largely child-led” nature at the technical level and aim to minimize the child’s preferences being subject to measurement errors caused by adult intervention or limitations in motor skills.

Beyond these technical design decisions, the tool’s theoretical foundation points in the same direction. The invisible integration of digital technologies into children’s daily lives (Plowman, 2016) and the emergence of children as a full-fledged “app generation” (Stratigos and Fenech, 2021) underscore the need for a measure that lets children express their responses independently of adult presence. Critical Constructivism offers a theoretical basis for this: children are seen as independent decision-makers (agency) rather than passive users of digital environments (Nolan et al., 2022; Wilson et al., 2024). The autonomy the digital interface affords is thus not only a pedagogical choice but also a means of obtaining less biased data. Building on this rationale, the tool was designed so the adult sits where they cannot see the child’s responses, reducing the chance that the child’s choices are shaped by the adult’s reactions or by a wish for approval. This is expected to partially, though not fully, reduce the tendency toward social desirability observed in the pen-and-paper format (Miller et al., 2015); the child may still sense that they are being assessed, so the tablet’s autonomy is a relative rather than absolute advantage.

Testing this expectation requires comparing the pen-and-paper version (administered under adult supervision) with the tablet version at two complementary levels: whether the two formats capture the same underlying construct (expected to show a strong positive correlation between scores) and whether (tested via the mean difference between administrations). Because adult supervision may increase the motivation to seek social approval (Miller et al., 2015; Di Norcia et al., 2018), scores on the tablet-based application were expected to differ from those on the paper-and-pencil format. This study tests that expectation descriptively, without examining the underlying causal mechanism.

The literature links temperament (particularly activity level and reactivity) to children’s risk-taking behaviors: children with high activity levels tend to prefer physically riskier play, while highly reactive children may respond to risky situations with either more caution or more variable reactions (Nystrom and Bengtsson, 2016). This relationship makes it necessary to test whether the assessment tool evaluates children with different temperament profiles with the same structure and precision; otherwise, a low score from a highly active child might reflect the tool functioning differently for that group rather than an actual low risk-taking tendency. Because the way children interact with a digital tablet may also vary by gender or temperament, the tool needs to demonstrate measurement invariance across both dimensions for the resulting scores to be meaningful and fair in cross-child comparisons. This study therefore aims to transform the WRiPS-P (originally a paper-and-pencil assessment) into a validated, reliable digital tool that children can complete independently, revealing their risk-taking tendencies free from adult supervision.

In line with this general objective, the following questions will be addressed:

1. What is the evidence for the construct validity of the developed tablet-based assessment tool in measuring risky play tendencies in preschool children?
2. What is the evidence for the measurement invariance of the developed tablet-based assessment tool across gender in measuring risky play tendencies in preschool children?
3. What is the evidence for the measurement invariance of the developed tablet-based assessment tool across temperament (activity level and reactivity) in measuring risky play tendencies in preschool children?
4. What is the evidence of reliability, in terms of internal consistency, for the tablet-based assessment tool that was developed in measuring risky play tendencies among preschool children?
5. Is the relationship between the developed tablet-based assessment tool and the paper-and-pencil version of the tool statistically significant?
6. Is there a statistically significant difference between the scores on the developed tablet-based assessment tool and the paper-and-pencil version of the tool?

## 2 Method

### 2.1 Research model

This study is a methodological investigation conducted within the framework of a descriptive-correlational research approach, aimed at testing the construct validity (confirmatory factor analysis) and measurement invariance across gender and various temperament profiles of a tablet-based assessment tool (Fraenkel et al., 2012). As part of the study, evidence of validity and reliability was collected for a test originally developed in a paper-and-pencil format, adapted for use as a tablet-based application.

### 2.2 Study group

For the purposes of this study, preschool children aged 5–6 were selected as the study group. Both convenience sampling and maximum diversity sampling methods were used to recruit participants. At the school level, maximum variation sampling was applied by selecting schools from lower-middle, middle, and upper-middle SES neighbourhoods in Kocaeli province; within these areas, accessible schools were then selected via convenience sampling. At the family level, variation was targeted across four characteristics, detailed in Table 1: maternal employment status, the child’s birth order (only child, eldest child, or younger sibling), parental age range, and gender balance. This approach aimed to ensure the study group reflected a broad range of early childhood play contexts. The demographic characteristics of the study group are presented in Table 1.

**Table 1:** The demographic characteristics of the study group.

Characteristics		*f*	%
Gender	Girls	102	51.5
	Boys	96	48.5
Birth order	Only child	19	9.6
	Eldest child	74	37.4
	Others	105	53.0
Mother’s age	25–35	90	45.5
	36–45	103	52.0
	45 and older	5	2.5
Father’s age	25–35	64	32.3
	36–45	114	57.6
	45 and older	20	10.1
Mother’s employment status	Employed	79	39.9
	Not employed	119	60.1
Father’s employment status	Employed	190	96.0
	Not employed	8	4.0

As shown in Table 1, the participant group exhibits a balanced distribution by gender (51.5%, *f* = 102 girls; 48.5%, *f* = 96 boys), and the majority come from families with more than one child (88.4%, *f* = 179). The majority of parents are between the ages of 25 and 45 (97.5%, *f* = 193 for mothers; 80.9%, *f* = 178 for fathers), and there is a marked difference between mothers and fathers in terms of employment status [only 39.9% of the mothers are employed (*f* = 79) while 96.0% of the fathers are employed (*f* = 190)]. The proportion of non-working mothers is significantly higher than that of fathers.

### 2.3 Data collection tool

#### 2.3.1 Demographic information form

This form, created by researchers, includes questions designed to determine the children’s gender, birth order, and the ages and employment status of their parents.

#### 2.3.2 Digitized willingness to risk in play scale – pictorial

The Digitized Willingness to Risk in Play Scale – Pictorial (d-WRiPS-P) is a digital adaptation of The Willingness to Risk in Play Scale: A Pictorial Scale for Preschool Children (WRiPS-P) (Ozyeter et al., (in review)). TheWRiPS-P is a pictorial assessment tool developed to directly evaluate the internal willingness to take risks during play among 5–6-year-old preschool children, based on Sandseter’s (2007) categories of risky play. It consists of a 4-point Likert scale (Level 0: “No willingness to take risks”–Level 3: “High desire to take risks”). To minimize gender and ethnic biases, the scale features a cute cartoon cat character, allowing children to express their emotional risk preferences without being hindered by language barriers. As a result of Exploratory Factor Analysis (EFA) and Confirmatory Factor Analysis (CFA), a three-factor structure comprising 15 items that explains 48.88% of the total variance (disappearing/distancing, physical activity, and high speed/dangerous tools)was confirmed; model fit indices confirmed the validity of the structure [*χ*^2^(87) = 99.467, *p* > 0.05; RMSEA = 0.031; CFI = 0.975; TLI = 0.970; SRMR = 0.058]. While content validity was established with nearly perfect inter-rater agreement (Cohen’s Kappa = 0.93), criterion-related validity analyses revealed positive and significant correlations between the Temperament Scale and the subdimensions of activity (*r* = 0.299), closeness/harmony (*r* = 0.314), and sensory sensitivity (*r* = 0.466). Cronbach’s alpha was calculated separately for the EFA and CFA datasets. Total score coefficients demonstrated good reliability at 0.80 and 0.81, respectively.

In the d-WRiPS-P, the three-factor structure of the WRiPS-P (“disappearing/distancing,” “physical activity,” and “high speed/dangerous tools”) was preserved exactly; only the mode of administration was changed, from a paper-and-pencil format to a tablet-based, audio-narrated, self-paced interface that the child completes independently, without adult intervention (see Section 1.1 for the interface design features and rationale). The tablet-based version automatically scores responses, supporting standardization and practicality in data collection and analysis.

#### 2.3.3 Temperament scale for children

The study utilized the Temperament Scale for Children developed by Özyürek et al. (2020). The scale is designed to assess the temperament traits of children aged 4–8 based on maternal reports. Rated on a five-point Likert scale (“never,” “rarely,” “sometimes,” “often,” “always”), the scale consists of a total of 33 items and 7 subscales. A high score on the scale indicates that the relevant temperament trait is dominant in the child. The subscales comprising the scale are as follows: (1) Activity Level, which measures the child’s motor activity during activities; (2) Affinity and Adaptability, which measures the level of adaptation to new people and situations; (3) Sensory Sensitivity, which measures the intensity with which emotions such as anger and fear are expressed at a normal level; (4) Reactivity, which measures responses to sensory stimuli such as sound, light, and smell; (5) Attention and Perseverance, which measures the ability to sustain attention and effort during activities; (6) Rhythmicity, which measures the regularity of biological rhythms such as sleep and hunger; and (7) Emotional Sensitivity, which measures the child’s sensitivity to the emotions of people in their environment. In this study, the reactivity and activity level subscales were used.

According to the evidence regarding the construct validity of the scale, the scale explains 57.20% of the total variance with 33 items grouped into 7 subscales. As part of the reliability analyses, the Cronbach’s alpha internal consistency coefficient calculated for the entire scale was found to be 0.85; the reliability coefficients for the subscales ranged from 0.57 to 0.82. In this study, the Cronbach’s alpha calculated for the Activity Level subscale in the original scale was 0.79, and for the Reactivity subscale, it was 0.82. These findings indicate that the items included in the scale possess strong psychometric qualities for distinguishing children’s temperament traits.

Standardized factor loadings were examined in the DFA models tested for the activity and reactivity subscales of the temperament scale. Factor loadings for the activity subscale ranged from 0.484 to 0.887, while those for the reactivity subscale ranged from 0.434 to 0.852. The fit indices for the Activity subscale are as follows: chi-square/df = 14.822/5, RMSEA = 0.10 [90% CI 0.043–0.160], CFI = 0.980, TLI = 0.959, SRMR = 0.039. With the exception of the RMSEA, all indices for the Activity subscale indicate excellent fit. The RMSEA, however, indicates a borderline acceptable fit. The fit indices calculated for the Reactivity subscale are chi-square/df = 20.753/8, RMSEA = 0.09 [90% CI 0.043–0.138], CFI = 0.992, TLI = 0.985, and SRMR = 0.023. A similar situation applies to the measurement model for the Reactivity subscale. All indices except RMSEA indicate excellent fit. RMSEA indicates an acceptable fit that is very close to the threshold.

### 2.4 Data collection

To collect the data, an application was initially submitted to the Kocaeli Provincial Directorate of National Education to obtain ethics committee approval (Kocaeli University Ethics Committee, protocol no: E-94094268-100-795447 and date: 19.06.2025) and to identify the appropriate study group in line with the research objectives. Contact was made with the administrators of the 12 preschool institutions authorized by the Provincial Directorate of National Education to participate in the study. The ethics committee approval, the authorization letter from the Directorate of National Education, and the purpose of the study were shared with the school administrators. Through the school administrators, participation consent forms were sent to parents via a digital platform. The digital form included a demographic information form for parents who consented to the study and a temperament scale for the children. The children of parents who consented to the study and their respective schools were listed, and a suitable sequence for the interventions was established. Since there were no volunteer participants at 4 of the listed schools, the study was conducted at 8 schools. At each of these 8 schools, the data collection process was completed with a minimum of 20 and a maximum of 32 children.

In this study, conducted as part of a Scientific Research Project, the data collection process was managed by one of the researchers, and the fieldwork was carried out by a scholarship recipient pursuing a master’s degree in the Department of Guidance and Psychological Counseling. Prior to data collection, a standardized training process was conducted to ensure the scholarship recipient’s competence in administering the interventions. First, while the scholarship recipient served as an observer, the researcher administered the intervention to 5 children; subsequently, the scholarship recipient administered the intervention to 5 children. During and after these sessions, the researcher and the scholarship recipient agreed on the most appropriate procedure for the intervention. The 10 sessions conducted for the scholarship recipient’s training were not included in the data.

The sessions were conducted in the guidance counseling office at the schools, which were quiet and free of distractions. In a setting equipped with a table and two chairs appropriate for the child’s height, the facilitator sat directly across from the child. The tablet was placed horizontally on the table in front of the child. Before the session, a 3–5-min conversation took place between the researcher and the child to get acquainted and help the child feel at ease. The researcher asked the child if they were willing to participate in the study and reminded them that they could stop at any time. The session then began with standard instructions explaining the assessment tool, and it concluded with a thank-you to the child.

For convergent validity with the WRiPS-P, the parents of the 50 children who had completed the tablet-based application were contacted again through the school administration, and consent was obtained for a new application. Thirty-five parents provided consent for their children’s participation. Provided that at least 4 weeks had elapsed since the initial application, the WRiPS-P was administered to these 35 children this time. The same procedure followed during the tablet-based measurement process was applied during this administration. The data from these 35 children were not included in the validity and reliability data of the measurement tool.

“*The digital version of the d-WRiPS-P is available as Supplementary material. Instructions for importing and using the survey are provided in the Supplementary file.*”

### 2.5 Data analysis

Various statistical methods were employed within the scope of the study to address the research questions.

Confirmatory factor analysis (CFA) was conducted to establish validity evidence for the d-WRiPS-P. Before performing this technique, its underlying assumptions were examined. These assumptions include an adequate sample size (MacCallum et al., 1999; Wolf et al., 2013) and a linear relationship between the observed variables and the latent factors (Brown, 2015; Kline, 2011). While the assumption of multivariate normality is important for continuous data, this assumption is not expected to be met for categorical/Likert-type data; for such data, it is recommended to use estimation methods suited to categorical data, such as WLSMV (Finney and DiStefano, 2013; Flora and Curran, 2004). For sample size, it is recommended that the number of observations be 3 to 10 times the number of items. In this study, data were collected from 198 participants for the 15-item scale. Scatter plots were examined to assess linearity. Since the scale was rated on a 4-point Likert-type format, WLSMV was selected as the estimation method. This method does not require the assumption of normality. Reliability evidence for the scale was calculated using the Ordinal Omega reliability coefficient in R version 4.3.2 (R Core Team, 2024) with semTools (Jorgensen et al., 2022) and psych package (Revelle, 2024).

Measurement invariance analyses were conducted to determine whether the d-WRiPS-P used in the study exhibits equivalent psychometric properties according to gender and children’s temperament characteristics (low reactivity/high reactivity, low activity/high activity). Children were ranked from the highest to the lowest score based on their total scores on the activity and reactivity subscales of the temperament scale. The Activity Level subdimension refers to the amount of movement, energy, and physical activity in the child’s daily life, that is, how active, restless, or calm the child’s disposition is. The Reactivity subdimension, on the other hand, refers to the intensity and strength of the emotional and behavioral response the child exhibits to the stimuli they encounter (e.g., frustration, sudden changes, disturbing situations), that is, how easily or intensely the child reacts. To identify students with low and high characteristics in the groups, upper and lower 27% groups were formed. Students in the upper 27% group were referred to as children with a high activity/reactivity level, and children in the lower 27% group were referred to as children with a low activity/reactivity level.

Measurement invariance involves a series of hierarchical model comparisons that test whether a measurement instrument measures the same construct with the same meaning and at the same level across different groups (Meredith, 1993; Vandenberg and Lance, 2000). In this study, invariance was tested at four sequential levels: configural, metric/weak, scalar/strong, and strict/residual (Putnick and Bornstein, 2016). In the first stage, a configural model was established that tests whether the same factor structure is confirmed in both groups without imposing any equality constraints. In the second stage, the metric invariance model, in which factor loadings are constrained to be equal across groups, was tested; achieving this level indicates that the items are associated with the latent factor with the same strength in each group. In the third stage, the scalar invariance model was examined, in which, in addition to the item loadings, the thresholds were also constrained to be equal across groups. Achieving invariance at this level indicates that the scores obtained from the scale are comparable. Finally, the strict invariance model, in which the item residual variances were also equated across groups, was tested; achieving this level indicates that the measurement error is equivalent across groups as well.

Since the data were categorical (Likert-type) in structure, all models were analysed in Mplus 8.3 using the WLSMV (Weighted Least Squares Mean and Variance adjusted) estimation method and THETA parameterization (Muthén and Muthén, 1998–2017; Flora and Curran, 2004). Comparisons between nested models were carried out using the corrected chi-square difference test (DIFFTEST) specific to WLSMV estimation (Muthén et al., 1997). The absence of a significant difference between models (*p* > 0.05) was interpreted as indicating that the higher-level constraint fit the model, that is, that the relevant level of invariance was achieved. In addition, due to the limitations of the chi-square test, which is sensitive to sample size, the ΔCFI value was used as a complementary criterion in model comparisons; a ΔCFI ≤ 0.010 was accepted as evidence that the relevant level of invariance was also supported in practical terms (Chen, 2007; Cheung and Rensvold, 2002). In evaluating model fit, values of CFI ≥ 0.90, TLI ≥ 0.90, RMSEA ≤ 0.10, and SRMR ≤ 0.08 were considered acceptable fit criteria (Browne and Cudeck, 1993; Hu and Bentler, 1999).

In the fifth research question, the relationship between the WRiPS-P scores and the d-WRiPS-P scores was examined using the Pearson Product–Moment Correlation Coefficient. This technique is applied to continuous variables that show a normal distribution. The total scale scores indicate that the variable is continuous. To examine normal distribution, skewness and kurtosis coefficients were examined. For the paper-pencil application scores, skewness was calculated as 0.792 and kurtosis as 0.205; for the scores obtained from the tablet-based application, skewness was calculated as 0.318 and kurtosis as −0.691. Since it has been stated that a distribution can be considered normal when the skewness and kurtosis coefficients fall within the ±1.5 limits (Tabachnick and Fidell, 2013), it was concluded that the scores obtained from both applications showed a normal distribution, and therefore it was appropriate to examine the relationship between the variables using the Pearson Product–Moment Correlation Coefficient. Since correlation analysis reflects the relative ranking (association) of the scores obtained from the two applications but does not capture systematic differences at the mean level (Bland and Altman, 1986), it was preferred to test the relationship between the two applications separately at both the relative (correlation) and absolute (mean difference) levels. In the final research question of the study, the statistical significance of the difference between the WRiPS-P and d-WRiPS-P scores was examined using the Paired Samples t-test. According to the assumptions of this technique, the variable is continuous. As a result of examining the normality of the difference scores, the difference scores showed a normal distribution (skewness = −0.246, kurtosis = −0.484).

## 3 Results

### 3.1 Digitized willingness to risk in play scale – pictorial (d-WRiPS-P) CFA results (RQ1)

The first psychometric property examined for the d-WRiPS-P is construct validity. The CFA model and fit indices obtained for this purpose are presented in Figure 1.

**Figure 1:**
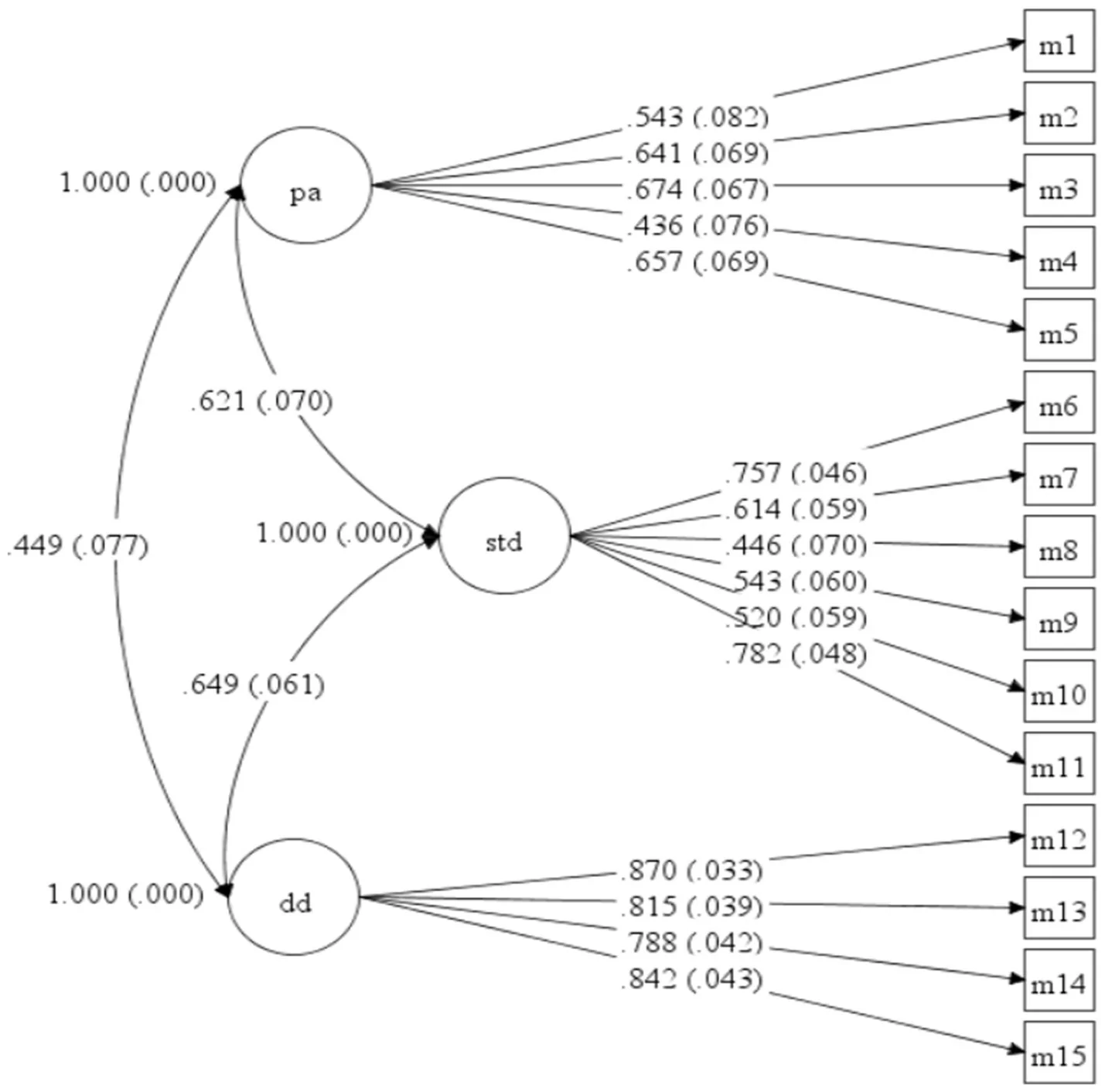
The CFA model and fit indices of d-WRiPS-P. PA = physical activity; SDT = speed and dangerous tools; DD = disappearing/distancing.

When the model presented in Figure 1 is examined, it is seen that the standardized factor loadings of the items range from 0.44 to 0.67 in the physical activity subdimension, from 0.45 to 0.78 in the speed and dangerous tools subdimension, and finally from 0.79 to 0.87 in the disappearing/distancing subdimension. The model fit indices are as follows: *χ*^2^/df = 128.87/87; CFI = 0.972; TLI = 0.966; RMSEA = 0.049; SRMR = 0.054.

To evaluate model fit in greater detail, modification indices (with a minimum M. I. threshold of 10; Muthén and Muthén, 1998–2017) were examined; this threshold is based on the fact that the M.I. follows an approximate *χ*^2^(1) distribution and that M. I. ≥ 10 corresponds to significance at the *p* < 0.001 level (Sörbom, 1989). Additionally, standardized residuals (SRMR) were examined. As a result of the analysis, only three modification suggestions were identified—all related to item M14—at a limited level (M. I. = 10.38–11.07) and of low magnitude (StdYX E. P. C. = 0.28–0.45); since none of these suggestions provided a theoretically meaningful justification based on cross-loading or error covariance, no post-hoc model changes were made. Furthermore, the SRMR value (0.054), which indicates the model’s overall residual fit, remained below the accepted threshold of 0.08, supporting that the model fits the data adequately (Hu and Bentler, 1999).

### 3.2 Measurement invariance findings by gender (RQ2)

Another method of gathering evidence for the validity of the scores obtained from the d-WRiPS-P is the measurement invariance test. The results of the multi-group measurement invariance test conducted to examine whether the d-WRiPS-P produces different results according to children’s gender are presented in Table 2.

**Table 2:** Measurement invariance for gender.

Model	*χ* ^2^	df	*p*	RMSEA [90% CI]	SRMR	TLI	CFI	Δ*χ*^2^	Δdf	*p*	ΔCFI
Configural	227.802	174	0.0038	0.056 [0.033–0.075]	0.077	0.949	0.957	–	–	–	–
Metric	239.870	186	0.0047	0.054 [0.031–0.073]	0.079	0.953	0.958	15.103	12	0.2358	0.000
Scalar	259.566	213	0.0161	0.047 [0.022–0.066]	0.080	0.964	0.964	22.177	27	0.7283	0.006
Strict	314.029	231	0.0002	0.060	0.090	0.941	0.935	34.454	15	0.0029	−0.029

Model comparisons were conducted sequentially.

The fit indices and model comparisons for the configural, metric, scalar, and strict invariance models which were tested sequentially, are presented in Table 2. The configural, metric, and scalar models showed good fit; the chi-square difference tests between these three models were found to be non-significant, and the ΔCFI values remained below the 0.01 criterion proposed by Cheung and Rensvold (2002). These findings indicate that the scale ensures partial measurement invariance (specifically supporting configural, metric, and scalar invariance) across genders; that is, the factor structure, item loadings, and item intercepts are equivalent between girls and boys. However, because strict (residual) invariance was not supported due to a significant deterioration in fit when error variances were equated [Δ*χ*^2^(15) = 34.454, *p* = 0.0029; ΔCFI = −0.029], the instrument cannot be assumed to possess full (strict) invariance across gender.

### 3.3 Measurement invariance findings by temperament (RQ3)

It was tested whether the scores obtained from the d-WRiPS-P function equivalently for children who like activity and those who do not, as well as for reactive and non-reactive children. Table 3 presents the measurement invariance findings for activity.

**Table 3:** Measurement invariance results for activity.

Model	*χ* ^2^	df	*p*	RMSEA [90% CI]	SRMR	TLI	CFI	Δ*χ*^2^	Δdf	*p*	ΔCFI
Configural	233.171	174	0.0018	0.080[0.050–0.106]	0.103	0.893	0.911	–	–	–	–
Metric	247.185	186	0.0018	0.079 [0.050–0.104]	0.106	0.896	0.908	17.130	12	0.1448	−0.003
Scalar	272.690	213	0.0036	0.073 [0.043–0.097]	0.108	0.912	0.910	28.538	27	0.3836	0.002
Strict	285.560	231	0.0084	0.067 [0.036–0.091]	0.115	0.925	0.918	15.210	15	0.4364	0.008

According to the findings presented in Table 3, acceptable fit indices were obtained for all hierarchically tested configural, metric, scalar, and strict models for both the high- and low-activity groups. The fact that the chi-square difference tests in the stepwise model comparisons were not significant and that all ΔCFI values remained below the recommended 0.01 critical threshold (Cheung and Rensvold, 2002) indicates that the d-WRiPS-P ensures strict measurement invariance across activity temperament groups. This finding demonstrates that the factor structure, factor loadings, item intercepts, and error variances are equivalent in both groups.

The results of the multi-group confirmatory factor analysis showing whether the scores obtained from the d-WRiPS-P function equivalently for children with high and low reactivity are presented in Table 4.

**Table 4:** Measurement invariance results for reactivity.

Model	*χ* ^2^	df	*p*	RMSEA [90% CI]	SRMR	TLI	CFI	Δ*χ*^2^	Δdf	*p*	ΔCFI
Configural	214.007	174	0.021	0.066 [0.028–0.094]	0.093	0.960	0.960	–	–	–	–
Metric	219.559	186	0.0466	0.058 [0.008–0.087]	0.094	0.962	0.966	8.443	12	0.7496	0.006
Scalar	250.520	213	0.0398	0.058 [0.014–0.085]	0.097	0.963	0.962	34.991	27	0.1391	−0.004
Strict	263.155	231	0.0718	0.051 [0.000–0.079]	0.103	0.971	0.968	15.619	15	0.4078	0.006

As shown in Table 4, all four models tested for the reactive and non-reactive groups fit the data well. In sequential model comparisons, no chi-square difference was found to be statistically significant, and all ΔCFI values remained below the recommended critical threshold of 0.01 (Cheung and Rensvold, 2002). These findings indicate that the scale ensures strict measurement invariance across reactivity temperament groups; that is, the factor structure, item loadings, item-intercepts, and error variances are fully equivalent across groups.

### 3.4 Digitized willingness to risk in play scale – pictorial (d-WRiPS-P) reliability results (RQ4)

Reliability evidence for the d-WRiPS-P was examined using the Ordinal Omega reliability coefficient. Among the subscales, the Ordinal Omega calculated for the physical activity subscale was 0.67, for the speed and dangerous tools subdimension was 0.74, and for the disappearing/distancing subscale was 0.85. The Ordinal Omega reliability coefficient estimated for the total scale score was calculated as 0.90.

### 3.5 Results on the convergent validity between d-WRiPS-P and WRiPS-P (RQ5)

The convergent validity and mean score difference findings presented below were obtained from the same subsample of 35 children described in the Method section; therefore, these two analyses do not represent independent pieces of evidence but rather reflect two complementary aspects of the same dataset (the strength of the relationship and the difference in mean level). In the study, the relationship between the d-WRiPS-P and WRiPS-P scores was examined using the Pearson Product–Moment Correlation Coefficient. The results of the analysis revealed a positive, high, and statistically significant relationship between the d-WRiPS-P and WRiPS-P scores (*r* = 0.92; 95% CI [0.85, 0.96], *p* < 0.01).

### 3.6 Results on the differences between d-WRiPS-P and WRiPS-P scores (RQ6)

The statistical significance of the difference between the scores obtained from the d-WRiPS-P and the WRiPS-P was examined using the Paired Samples t-test. The findings are presented in Table 5.

**Table 5:** Difference test for d-WRiPS-P and WRiPS-P scores.

Measure	*n*	x̄	ss	*t*	sd	*p*	Cohen’s *d*	95% CI
WRiPS-P	35	38.06	7.01	4.196	34	0.000	0.71	[0.33; 1.08]
d-WRiPS-P	35	40.34	7.99					

95% CI for Cohen’s *d* was calculated using the noncentral *t* distribution method.

When Table 5 is examined, it is seen that the mean of the students’ scores on the WRiPS-P (X̄ = 38.06; SD = 7.01) was lower than the mean of their scores on the tablet-based application (X̄ = 40.34; SD = 7.99). As a result of the paired samples t-test, this difference was found to be statistically significant [*t*(34) = 4.196; *p* < 0.001]. The effect size calculated to assess the magnitude of this difference (*d* = 0.71; 95% CI [0.33, 1.08]) is, according to Cohen's (1988) criteria, above a medium level and close to a large effect. This indicates that the observed difference is of a considerable magnitude not only statistically but also practically.

## 4 Discussion

This study examined the psychometric properties of the d-WRiPS-P, the tablet-based version of the WRiPS-P, evaluating its construct validity, measurement invariance by gender and temperament, internal consistency, and convergent validity with the paper-and-pencil form. The tablet-based application proved to be a valid and reliable tool for assessing risky play tendencies in preschool-aged children.

The confirmatory factor analysis supported the scale’s three-factor structure (“physical activity,” “high speed and dangerous tools,” and “disappearing/distancing”) as established in the paper-and-pencil administration, confirming it in the tablet-based data. This is consistent with Sandseter’s (2007, 2009a) view that risky play consists of distinct yet complementary behavioral dimensions, and indicates that children’s risky-play tendencies can be assessed multidimensionally in both formats. The findings also align with the dimensions identified in the Risk-Taking in Play Scale (Ünüvar and Kanyılmaz, 2017) and the Tolerance for Risk-Taking in Play Scale (Hill and Bundy, 2014), both developed from Sandseter’s classification.

The items in the “disappearing/distancing” subdimension show higher and more homogeneous loadings than those in the other subdimensions, likely because “disappearance” behaviors represent a more consistent behavioral category; fear of separation from parents is developmentally typical in young children (Chiu et al., 2016), which may explain why these items converge on the same underlying structure. The physical activity subscale, by contrast, involves distinct motor skills such as climbing, running, and jumping (Gallahue and Ozmun, 2014), and early-childhood motor development varies considerably among children (Anderson et al., 2021); this heterogeneity likely reduced the common variance among items and, in turn, the subscale’s internal consistency (DeVellis, 2017; Hair et al., 2010).

The scale also demonstrated measurement invariance across configural, metric, and scalar levels for gender, indicating that it measures the same psychological construct similarly among girls and boys and that latent-mean comparisons between the groups are not affected by measurement bias. Strict invariance was not established, indicating that error variances are not exactly equal across groups; scalar invariance, however, is generally considered sufficient for comparing group means in the literature (Cheung and Rensvold, 2002; Chen, 2007; Putnick and Bornstein, 2016).

Measurement invariance was similarly supported for temperament: configural, metric, scalar, and strict invariance were established for both activity level and reactivity, indicating that the scale measures the same structure with the same sensitivity across children with different temperament traits and that risky-play tendency is not subject to an assessment bias specific to more active or more reactive children. High internal consistency for both traits further indicates similar measurement sensitivity across groups.

Reliability coefficients for the total score and the “disappearing/distancing” and “high speed/dangerous tools” subscales ranged from acceptable to high. The lower coefficient for the physical activity subscale likely reflects its more heterogeneous structure, which spans several distinct motor behaviors. Reliability coefficients also tend to be lower in subscales with fewer items. Taken together, these results indicate that the tablet-based scale produces sufficiently reliable measurements.

One of the study’s most significant contributions is the tablet-based application’s high alignment with the paper-and-pencil version. Because the convergent-validity finding (*r* = 0.92) and the mean-score difference (*d* = 0.71) came from the same sample of 35 children, they are best read as two complementary aspects of one comparison rather than independent pieces of evidence. The strong, significant correlation indicates that changing the assessment format does not alter the construct being measured, supporting the tablet-based application as a valid measurement method in its own right rather than merely a technological alternative.

Despite this strong relationship, average scores on the tablet were higher than on the paper-and-pencil version, with a statistically significant, moderate-to-large effect (*d* = 0.71). This difference is consistent with the expectation that adult supervision may increase children’s motivation to seek social approval, and that minimizing this pressure through a more autonomous tablet format might reflect higher, less-constrained self-reported scores (Miller et al., 2015; Di Norcia et al., 2018). The two formats appear to measure the same construct with the same factor pattern, but at different absolute levels, paralleling the configural/metric/scalar distinction used in the invariance analyses. An alternative explanation is that children found the tablet more game-like and were consequently more willing to take risks; the present data cannot distinguish between these two mechanisms, which future research should test directly through a measurement invariance analysis across formats. The direction of the difference also argues against a practice or familiarity effect: tasks were administered in a fixed order (tablet first, paper-and-pencil at least 4 weeks later), so familiarity would have been expected to raise (not lower) the second administration’s scores, the opposite of what was observed. This pattern instead may reflect variations associated with the administration mode itself, and can be read through the lens of Critical Constructivism and digital agency: the autonomy the interface affords children is consistent with their role as active decision-makers rather than passive participants, which may be reflected in their willingness to report higher risk preferences when unsupervised. As noted earlier, this autonomy is relative rather than absolute, so the underlying mechanism warrants further examination in future qualitative or experimental work.

## 5 Conclusion

In conclusion, this study provides evidence supporting the construct validity, measurement invariance, internal consistency, and convergent validity of the d-WRiPS-P. Specifically, while measurement invariance across temperament subgroups (activity and reactivity) was fully supported up to the strict level, gender invariance was supported only at the scalar level. Fit indices and factor loadings were consistent across these subgroups, confirming that while the scale is suitable for latent mean comparisons between genders, the measurement error variances are not fully equivalent across girls and boys. Despite this difference in residual variances, the reliability findings supported the total score. The physical activity subscale showed only moderate reliability, which is an acceptable trade-off given the instrument’s primary aim of yielding a total score. The positive, significant correlation between the tablet-based and paper-and-pencil scores indicates that the d-WRiPS-P is a valid measurement tool that can be used in place of, or alongside, the original paper-and-pencil version. Taken together, the findings show that the scale is a valid and reliable tool for assessing risky play tendencies in 5–6-year-old children, producing comparable results across sub-groups.

A further contribution of the study is that the digital format lets children participate in the assessment independently, without adult intervention; the systematic score difference between the tablet-based and paper-and-pencil formats is noteworthy in this regard, as it suggests that the mode of administration may be associated with variations in children’s responses, warrants further investigation. Because the tool relies on voice-over narration and a touchscreen interface, it also does not require literacy skills or adult assistance, though its applicability and measurement properties in populations where these conditions differ still need empirical testing. Although the tool was developed and evaluated in the Turkish context, its digital format and standardized administration procedures provide a foundation for future cross-cultural and multilingual comparative studies. From an implementation perspective, the tool’s short administration time, standardized instructions, and automatic scoring also address researchers’ and teachers’ need for consistent data collection independent of administrator error and time constraints.

## 6 Limitations and future research

The first limitation of the study is that it was conducted using a cross-sectional design. Additionally, while the study established strong internal consistency and construct validity, the temporal stability of the d-WRiPS-P (test–retest reliability) was not empirically evaluated, which remains a limitation. Future longitudinal and multi-wavestudies may reveal the developmental trajectory of the tendency towards risky play and the extent to which tablet-based assessment accurately captures this trajectory.

Although the study group met the criteria in the literature, larger groups are recommended in future validity and reliability research to reduce variance. This is particularly relevant to the temperament-based invariance analyses: the upper and lower 27% activity and reactivity groups (*n* = 53–54 each) were considerably smaller than the total sample, which may partly explain the relatively poor fit indices for activity-level invariance and calls for cautious interpretation of these findings. Testing measurement invariance across temperament groups with a larger total sample and at least 100–150 participants per group would strengthen future findings (Wolf et al., 2013).

The data were also collected from eight schools in a single province, selected through convenience and maximum-diversity sampling. Although the sample was designed to represent varied family and economic characteristics, its restriction to one province and its non-random selection limit the generalizability of the findings to children in other geographical, cultural, and socio-economic contexts. Larger, probability-sampled groups drawn from multiple regions would strengthen the representativeness of future findings.

Although the study aims to capture children’s intrinsic risk-taking intentions independent of their motor skills, digital scenarios cannot fully simulate physical action on a real playground. Given the ethical concerns of exposing children to real injury risk, this methodological choice is supported by existing research: Morrongiello (2004) found that children’s stated risk-taking intentions correlate highly with their actual behavior and serve as a valid proxy for it. The d-WRiPS-P addresses this limitation in ecological validity by focusing on safely and validly measuring the pre-action cognitive risk appraisal that precedes physical action, rather than the action itself.

A further limitation is that the temperament variable was assessed using only two of the scale’s seven sub-dimensions (activity level and reactivity) chosen for their strong association with risk-taking behavior in the literature (Nystrom and Bengtsson, 2016) and to keep the measurement invariance analysis focused. The possible effects of other temperament dimensions, such as sensory sensitivity or attention and perseverance, on the reliability of tablet-based assessment therefore fall outside this study’s scope; including them in future assessments could offer a fuller picture of how fairly the tool works across different temperament profiles.

Finally, the tablet-based format may limit the tool’s applicability in rural or socio-economically disadvantaged areas with limited access to digital infrastructure, a diversity the current sample does not represent. Testing the tool’s usability and measurement reliability with children from different socio-economic backgrounds and technological conditions would strengthen the generalizability of the findings and clarify its accessibility in early childhood settings.

## Data Availability

The datasets generated and analyzed during the current study are not publicly available because open repository consent was not obtained from the participants’ legal guardians. However, data are available from the corresponding author upon reasonable request for academic research purposes, subject to institutional ethical approval and a formal Data Use Agreement.
