# Supplementary material for: Through children’s own lenses: assessing risky play tendencies via a tablet-based interface
Source: Front Psychol. 2026 Sep 22;17:1951082. doi: 10.3389/fpsyg.2026.1951082 (PMC13638412; doi:10.3389/fpsyg.2026.1951082)
Supplement: Supplementary file 1 [file Supplementary_file_1.pdf]

Supplementary File 1

RESEARCH INSTRUMENT

The Willingness to Risk in Play Scale:

A Pictorial Scale for Preschool Children (WRiPS-P)

Administrator Instructions & Scoring Form

Administrator Instructions

The following administrator instructions will guide you in measuring a child's willingness to take risks in play. It is used with children aged 5–6. During administration, the scale pages should be used single-sided and in color. It is administered individually and face-to-face with each child. Please administer the scale individually, in a quiet setting free of distractions.

Before beginning the actual administration, the administrator should give the child the following instructions:

> *"We are going to play a picture game together. In this game, there is a cartoon character. This character is a kitten. When you look at these four pictures, which kitten would you want to be? I want you to show me that picture. There is no right or wrong answer here; I just want to know which kitten you would want to be. Here is an example of what you will do. Let's look at the pictures together."*

Character introduction picture

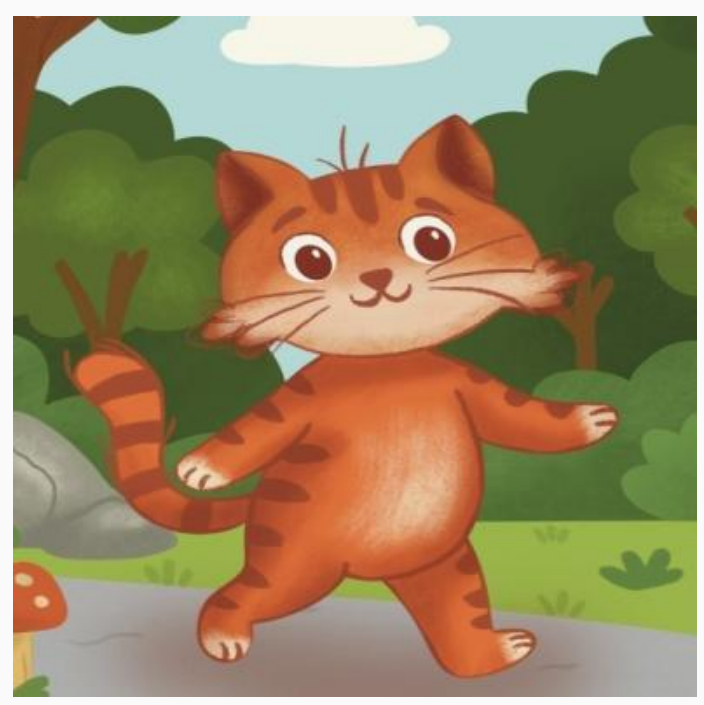

Example script2

Point with your finger to the first picture and say: "The kitten does not want to play the jumping-off-the-trampoline game." Point with your finger to the second picture and say: "The kitten wants to play the jumping-off-the-trampoline game while holding an older cat's paw." Point with your finger to the third picture and say: "The kitten wants an older cat nearby while playing the jumping-off-the-trampoline game." Finally, point with your finger to the fourth picture and say: "The kitten is playing the jumping-off-the-trampoline game alone."

Example item pictures (0–3)

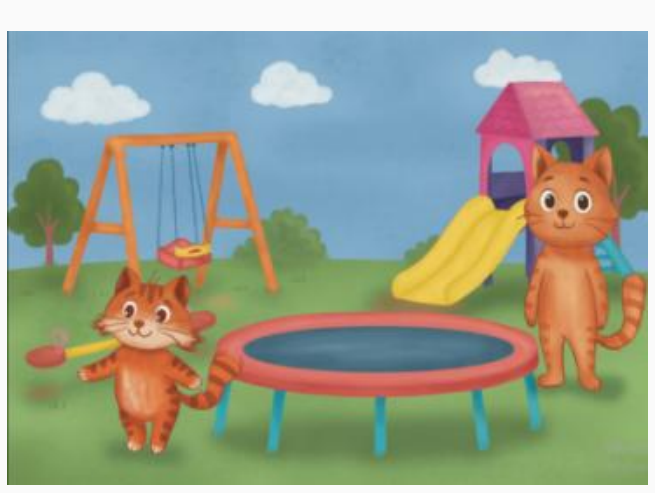
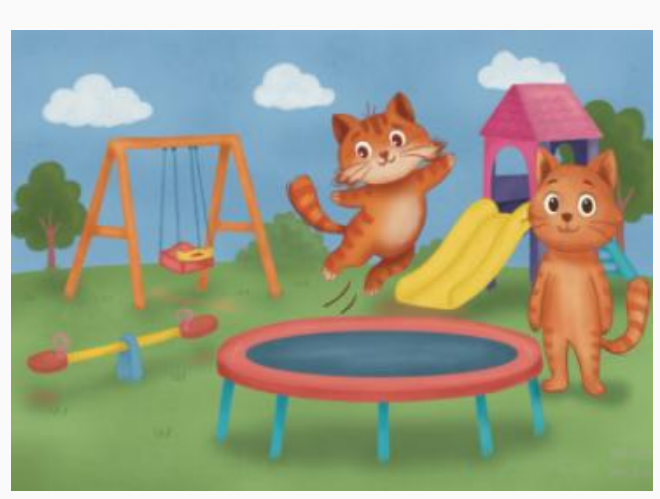
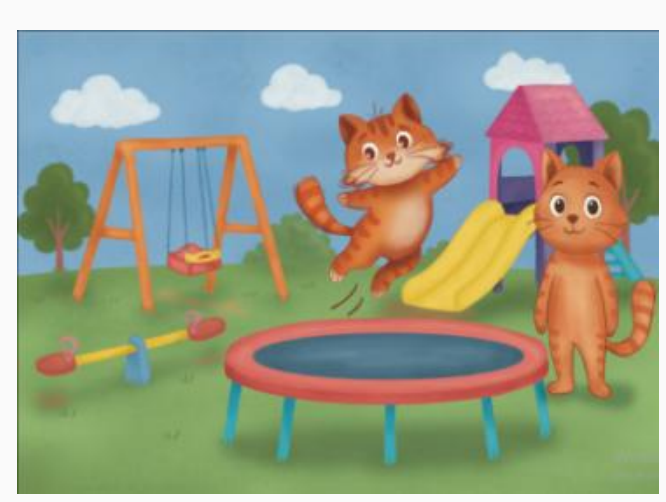
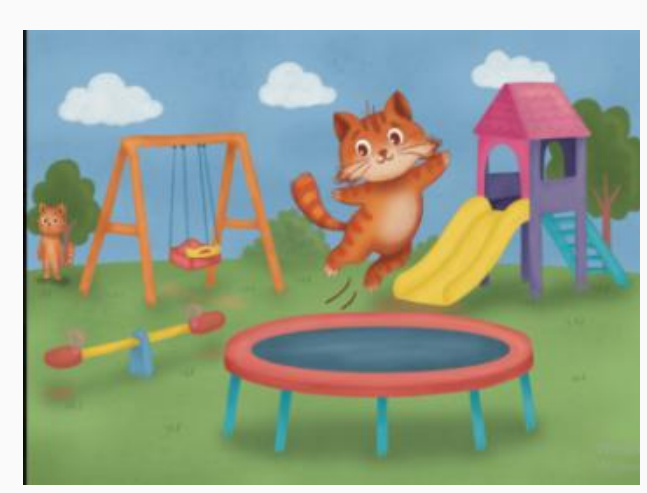

Ask the child, “When you look at these four pictures, which kitten would you want to be?” and say, “Please show me with your finger.” Finally, say, “Do you understand what you need to do now?” If the child says they understand, proceed to the actual administration.

During the actual administration, mark the number under the picture the child points to by circling it. Make sure you circle only one number for each item.

Scoring the Scale

Each item on the scale contains four pictures. Three pictures involve different levels of risk, while one picture involves no risk at all. A score of 0 indicates no willingness to risk; a score of 1 indicates a low level willingness to risk; a score of 2 indicates a medium level willingness to risk; and a score of 3 indicates a high level willingness to risk. If the child points to the first picture, it is understood that they have no willingness to risk; if they point to the second picture, a low level willingness to risk; if they point to the third picture, a medium level willingness to risk; and if they point to the fourth picture, a high level willingness to risk.

Willingness to risk	No willingness	Low level	Medium level	High level
—	0	1	2	3

The scale, *consisting of disappearing risk, risk associated with physical activity, and speed/risk of dangerous tools,* consists of a total of 15 items across three risk-in-play categories. The lowest possible score on the scale is 0, and the highest is 45. Children’s willingness to take risks in play is evaluated using the total score. The higher the score, the more willing the child is to take risks.

Scoring Chart

Child Number	Date of Administration
**Child's Date of Birth**	**Sex** ☐ **Girl** ☐ **Boy**
**Neighbourhood** ☐ Gated community ☐ Open neighbourhood	

For each item below, circle the single number that corresponds to the picture the child selected.

Risk Associated with Physical Activity

Item				
**1**	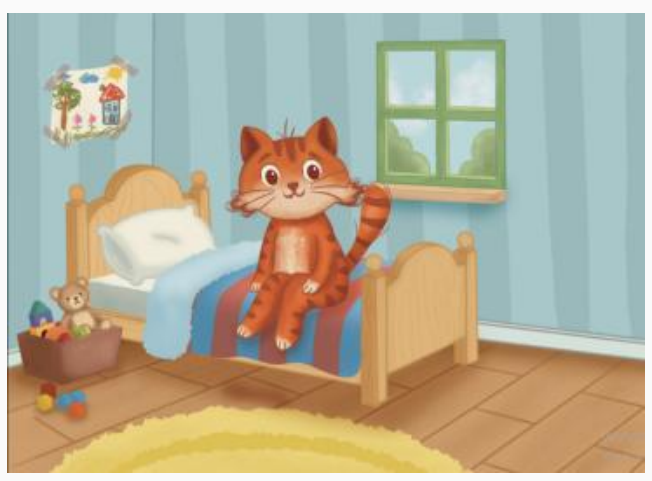	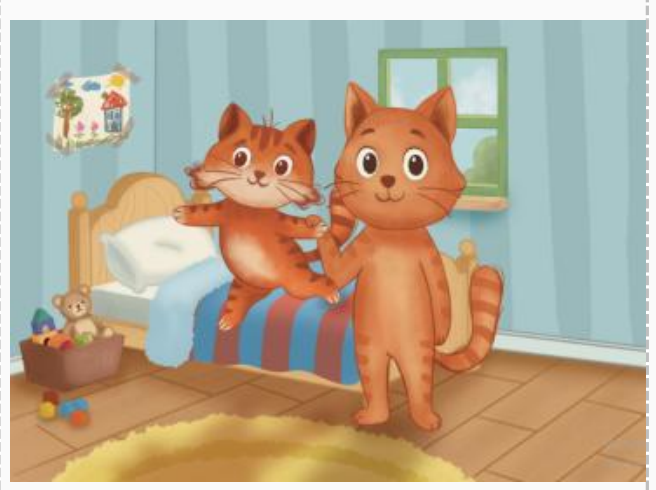	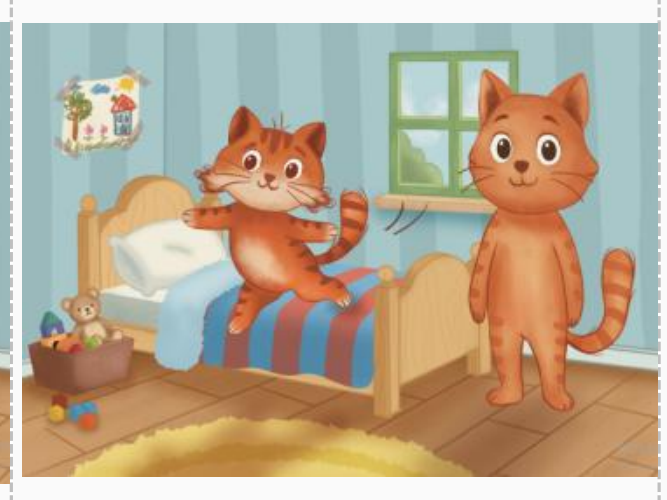	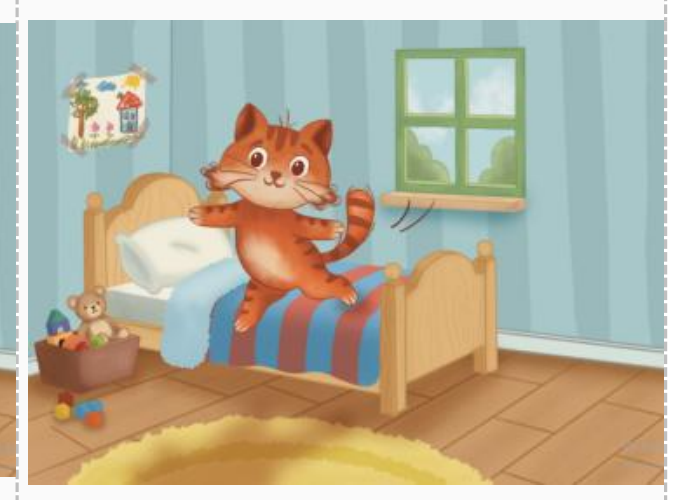
	The kitten does not want to play the jumping-off-the-bed game	The kitten wants to play the jumping-off-the-bed game while holding an older cat's paw	The kitten wants an older cat nearby while playing the jumping-off-the-bed game	The kitten is playing the jumping-off-the-bed game alone in its room
	**(0)** *No willingness to risk*	**(1)** *Low level willingness to risk*	**(2)** *Medium level willingness to risk*	**(3)** *High level willingness to risk*

Item				
**3**	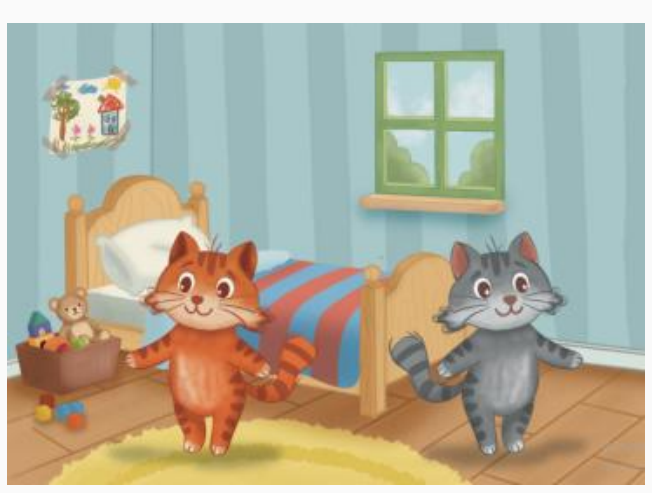	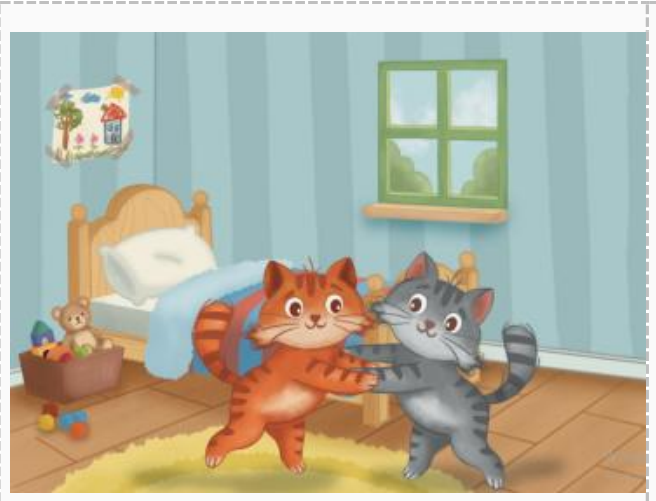	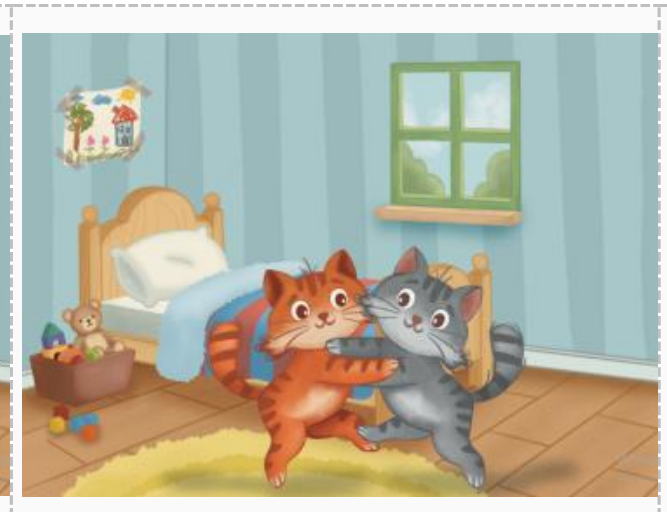	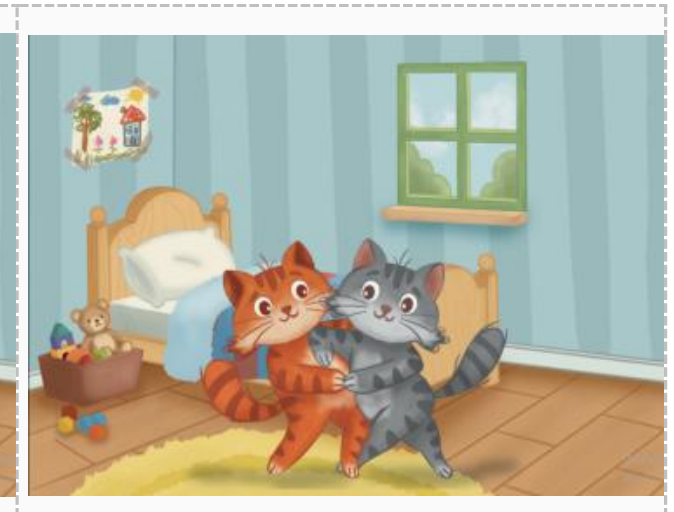
	The kitten does not want to play the wrestling game with a friend	The kitten is playing the wrestling game using only two legs	The kitten is playing the wrestling game using all of its legs	The kitten is playing the wrestling game with all its strength
	**(0)** *No willingness to risk*	**(1)** *Low level willingness to risk*	**(2)** *Medium level willingness to risk*	**(3)** *High level willingness to risk*

Speed / Risk of Dangerous Tools

**6**	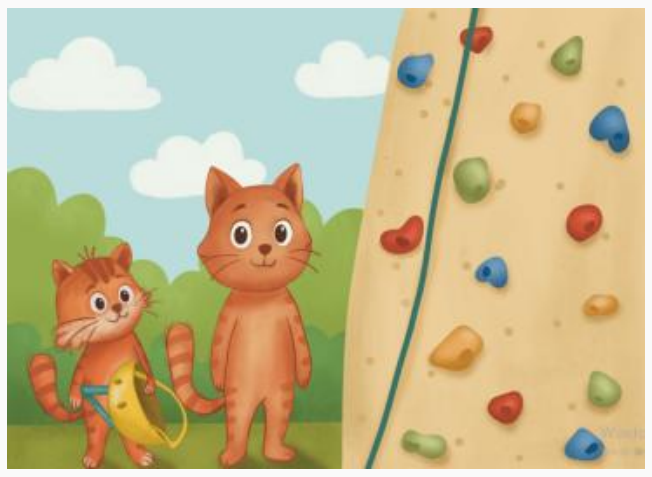	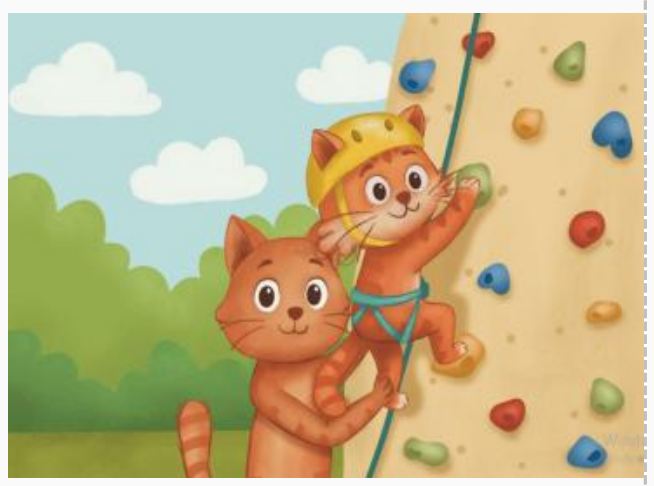	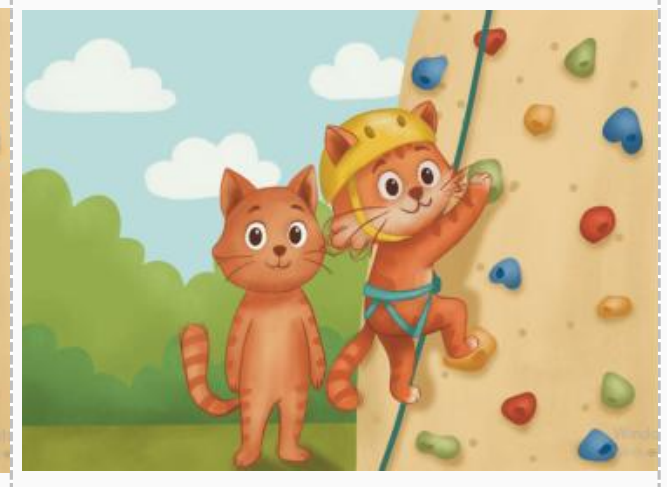	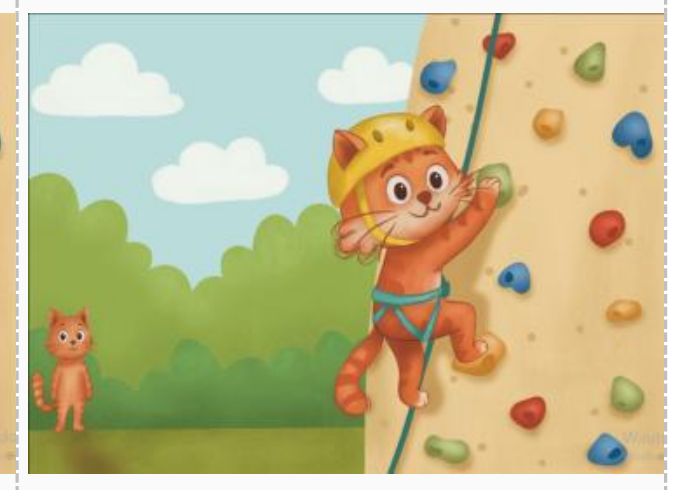
	The kitten does not want to climb the climbing wall	The kitten wants an older cat to hold it while climbing the climbing wall	The kitten wants an older cat nearby while climbing the climbing wall	The kitten is climbing the climbing wall alone
	**(0)** *No willingness to risk*	**(1)** *Low level willingness to risk*	**(2)** *Medium level willingness to risk*	**(3)** *High level willingness to risk*

**7**	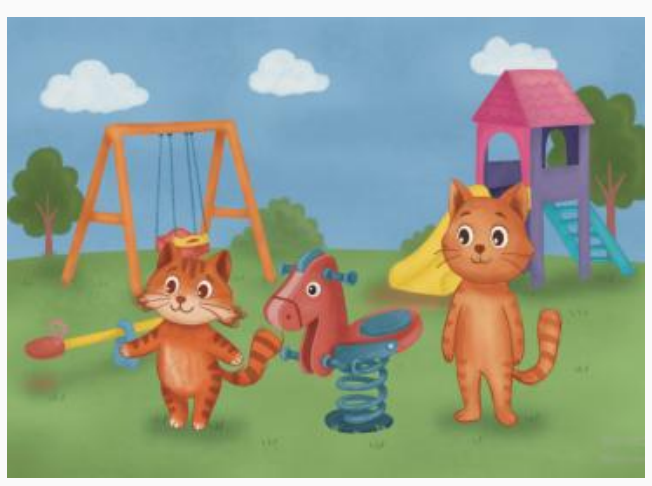	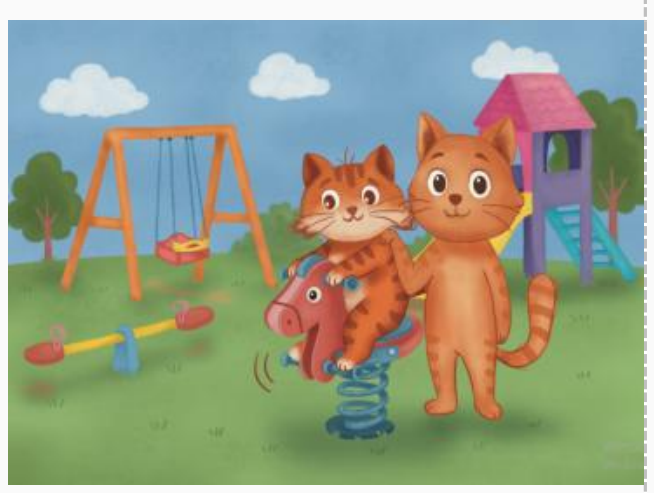	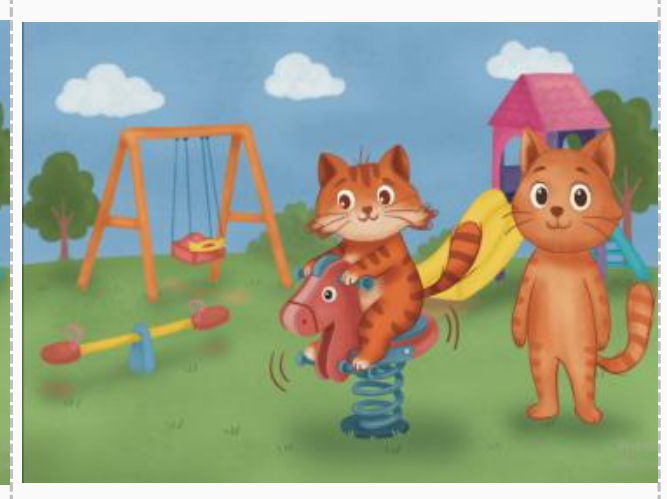	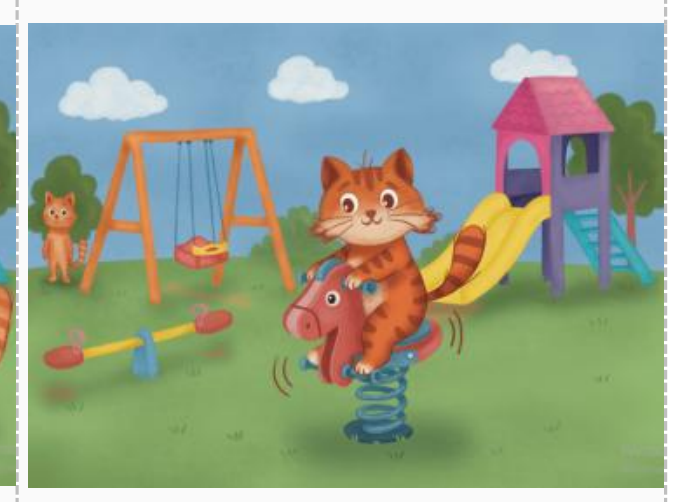
	The kitten does not want to play on the swinging toy at the park	The kitten wants an older cat to hold it while playing on the swinging toy	The kitten wants an older cat nearby while playing on the swinging toy	The kitten is playing on the swinging toy alone
	**(0)** *No willingness to risk*	**(1)** *Low level willingness to risk*	**(2)** *Medium level willingness to risk*	**(3)** *High level willingness to risk*

Disappearing

12				
	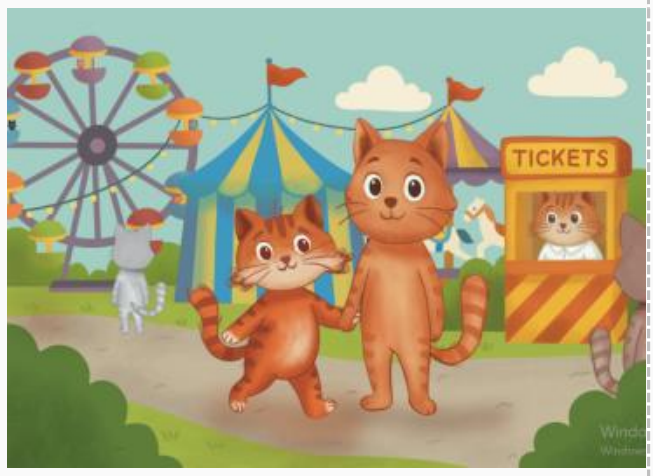	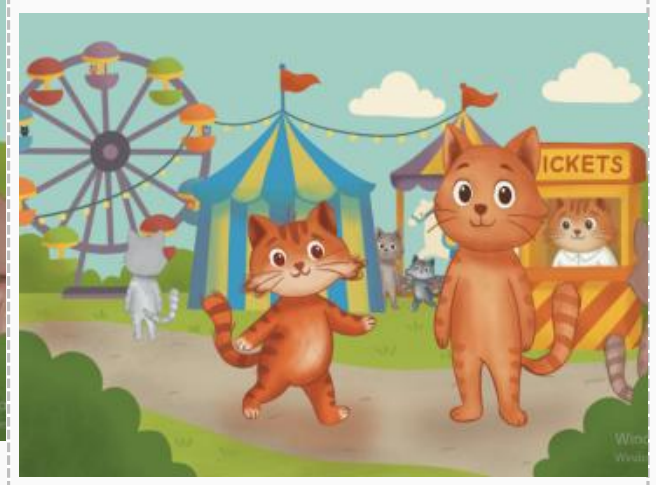	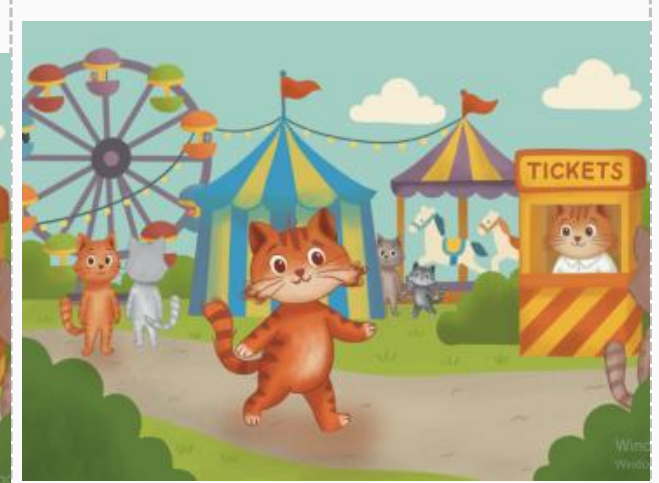	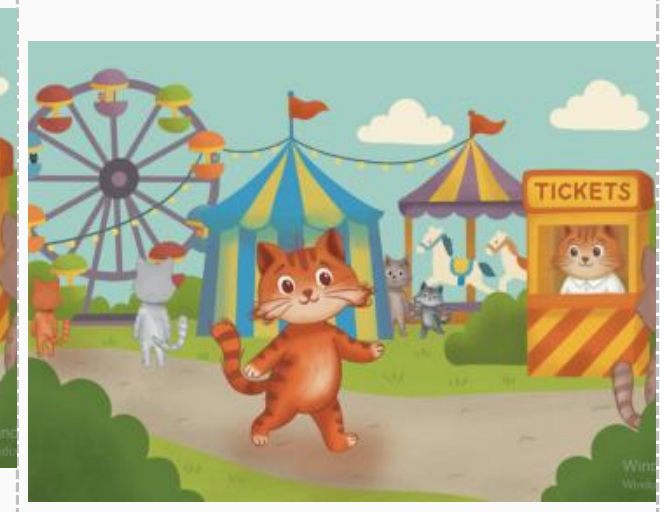
	The kitten is exploring the amusement park while holding an older cat's paw	The kitten is exploring the amusement park while an older cat is nearby	The kitten is exploring the amusement park while an older cat is at a distance but still in sight	The kitten is exploring the amusement park in a place where the older cat cannot see it
	**(0)**<br>*No willingness to risk*	**(1)**<br>*Low level willingness to risk*	**(2)**<br>*Medium level willingness to risk*	**(3)**<br>*High level willingness to risk*

13				
	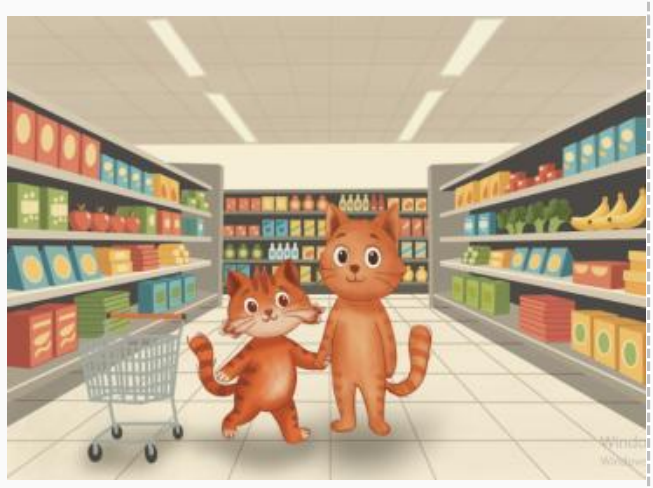	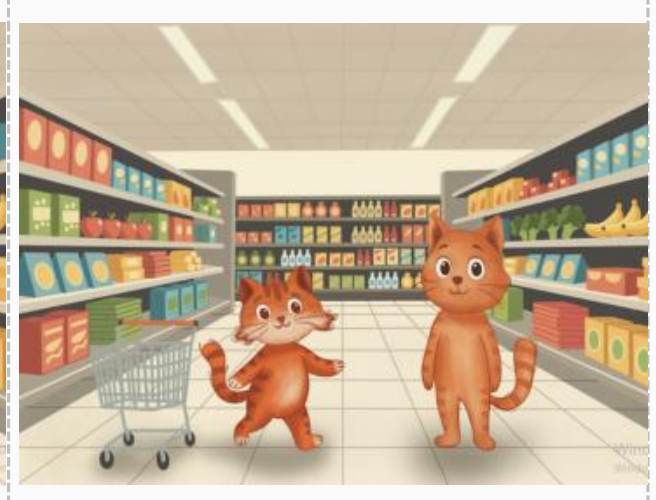	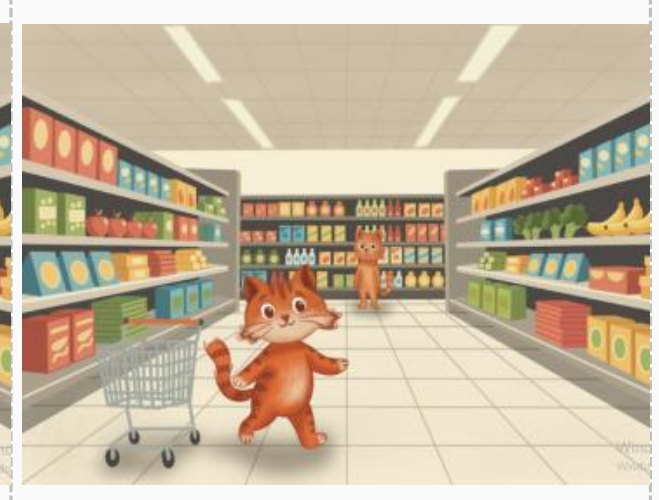	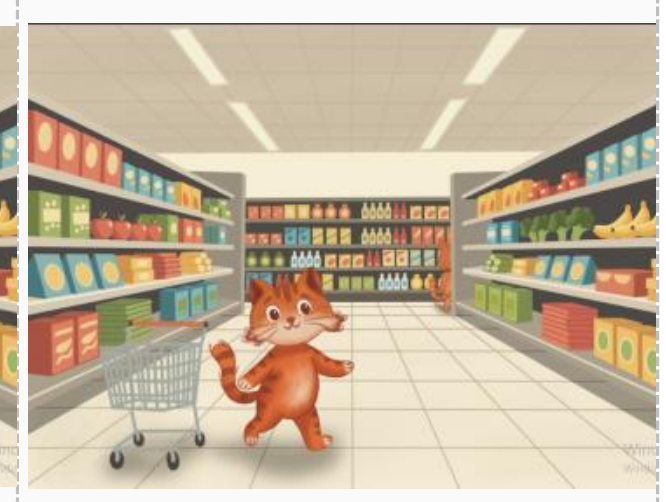
	The kitten is exploring the market while holding an older cat's paw	The kitten is exploring the market while an older cat is nearby	The kitten is exploring the market while an older cat is at a distance but still in sight	The kitten is exploring the market in a place where the older cat cannot see it
	**(0)**<br>*No willingness to risk*	**(1)**<br>*Low level willingness to risk*	**(2)**<br>*Medium level willingness to risk*	**(3)**<br>*High level willingness to risk*
